# Dietary Inclusion of Carob Pulp (*Ceratonia siliqua* L.) Does Not Replace the Antioxidant Effect of Vitamin E in Lambs’ Meat to Lengthen Shelf-Life

**DOI:** 10.3390/ani14243629

**Published:** 2024-12-16

**Authors:** Diego Nicolas Bottegal, Javier Álvarez-Rodríguez, María Ángeles Latorre, Sandra Lobón

**Affiliations:** 1 Departament de Ciència Animal, Universitat de Lleida, Av. Alcalde Rovira Roure 191, 25198 Lleida, Spain; javier.alvarez@udl.cat; 2 Instituto Nacional de Tecnología Agropecuaria (INTA), Rivadavia 1439, Buenos Aires C1033AAE, Argentina; 3 Departamento de Producción Animal y Ciencia de los Alimentos, Escuela Politécnica Superior, Universidad de Zaragoza, Carretera de Cuarte s/n, 22071 Huesca, Spain; 4 Departamento de Producción Animal y Ciencia de los Alimentos, Universidad de Zaragoza, Miguel Servet 177, 50013 Zaragoza, Spain; malatorr@unizar.es; 5 Instituto Agroalimentario de Aragón—IA2, CITA-Universidad de Zaragoza, 50059 Zaragoza, Spain; slobon@cita-aragon.es; 6 Departamento de Ciencia Animal, Centro de Investigación y Tecnología Agroalimentaria de Aragón (CITA), Av. Montañana 930, 50059 Zaragoza, Spain

**Keywords:** tocopherol, polyphenols, meat quality, condensed tannins, fatty acids

## Abstract

The objective of this study was to evaluate the impact of dietary carob pulp (Cp, *Ceratonia siliqua* L.) and vitamin E (Vit E) on the quality and shelf-life of light lamb meat stored for a maximum of 15 days under modified atmosphere packaging (MAP). Forty-eight lambs (half females and half entire males, 25.3 ± 0.3 kg of body weight and 83 ± 5.8 days old) were randomly selected and slaughtered after an experimental fattening period with a 2 × 2 factorial design: 0 vs. 20% Cp and 40 vs. 300 IU of Vit E/kg of diet for 41 days. The carcass traits, fatty acid (FA) profile, antioxidant content, colour, lipid oxidation, and microbial count in the meat were evaluated. Scarce interactions between Cp and Vit E were observed on most of the variables studied. The dietary inclusion of Cp did not affect carcass traits (*p* > 0.05) but produced minor changes in the content of branched FA and reduced the α-tocopherol content in the meat (*p* < 0.05), while no impact was observed on discolouration or lipid oxidation (*p* > 0.05). High Vit E supplementation increased the yellowness of caudal fat and the α-tocopherol content, which limited discolouration and lipid oxidation (*p* < 0.05). Furthermore, the High Vit E diet (without Cp) controlled the psychrotrophic bacterial count after 15 days of storage. At slaughter, males were heavier than females but had lower carcass dressing (*p* < 0.05). The lambs’ sex had minor impacts on the quality and shelf-life of meat. This study demonstrated that there is no synergistic interaction between Cp and Vit E on lamb meat quality and shelf-life. Including 20% of Cp in lamb diets is feasible without impairing meat quality. Nevertheless, supplementation with 300 IU of Vit E/kg of feed is essential to extend the shelf-life of meat stored under MAP.

## Simple Summary

In ruminant nutrition, the use of by-products, such as carob pulp which is rich in polyphenols, may be an alternative to traditional ingredients, providing additional antioxidant effects. This study examined the impacts of dietary carob pulp inclusion (0 vs. 20%) and vitamin E (40 vs. 300 international units/kg of feed) on the quality and shelf-life of lamb meat stored up to 15 days. The study found that neither carob pulp nor vitamin E significantly affected carcass traits. While carob pulp slightly altered fatty acid composition and reduced α-tocopherol in meat, it did not impact colour, lipid oxidation, or microbial count. High vitamin E supplementation increased α-tocopherol content, improving colour and reducing lipid oxidation. Additionally, high vitamin E diets contributed to control bacterial growth during storage. The lambs’ sex had minor effects on meat quality. Overall, the study concluded that 20% carob pulp inclusion in lamb diets is feasible, but high levels of vitamin E/kg of feed are necessary to extend shelf-life under modified atmosphere packaging.

## 1. Introduction

One of the most important challenges in the retail sector is to extend meat shelf-life and delay oxidative damage, reducing economic losses and food waste [1]. Lamb meat is especially susceptible to oxidation since it has more polyunsaturated fatty acids (PUFA) compared to beef [1]. During the storage time, oxidative spoilage depends on the balance between antioxidants and prooxidant elements (e.g., chelating trace elements, myoglobin and PUFA) in meat [2]. Thus, some of the most common strategies to reduce meat spoilage are adding antioxidants, such as tocopherols, carotenoids, and polyphenols, into animal feed or meat products [3], as well as using packaging technologies, such as modified atmosphere packs (MAP) [4] or vacuum packing [5].

Meat consumers demand more “natural” and healthier products, with a growing concern about the potential health hazards of synthetic antioxidants [6] driving research on alternative antioxidants, such as α-tocopherol or polyphenolic compounds, as possible substitutes for synthetic additives [7]. α-tocopherol is the most active isomer of the vitamin E (Vit E), which plays a vital role in scavenging reactive oxygen species (ROS). The ROS formed during retail display affect the bright red colour and induce the peroxidation of unsaturated fatty acids, developing an off-odour and off-flavour and decreasing the nutritional value of meat (due to accumulation of toxic compounds) [8]. Spanish nutritional guidelines recommend a dose of 40 IU of Vit E/kg of feed to prevent pathological conditions in lambs [9]. However, the inclusion of a high level of Vit E (more than 120 IU/kg of feed) in lambs’ diets, has been demonstrated to delay lipid oxidation and preserve colour stability, which extends the shelf-life [10,11], but increase the feeding cost. The Vit E dose and the length of the supplementation are important to achieve the optimal muscular deposit of Vit E and control meat deterioration [12]; however, both issues are not clearly stablished. 

On the other hand, polyphenols, such as phenolic acids, flavonoids (e.g., catechins and anthocyanidins), and hydrolysable and condensed tannins exhibit powerful antioxidant mechanisms scavenging ROS, chelating redox-active metals, and modulating endogenous defences [13,14]. Recent studies analysed the impact of including flavonoids in beef diets [15] or condensed tannins (CTs) in lamb diets [16] and reported minor changes in meat colour, but highlighted the increase in oxidative stability. Additionally, feeding ruminants with moderate levels of CTs may alter ruminal biohydrogenation, reflecting in changes in the fatty acid (FA) profile of milk or meat [17], although with variables results, due in part to the diversity of tannin types (molecular weight) and concentrations. The aforementioned effects have been studied, including up to 30% of carob pulp (Cp), a Mediterranean by-product, in lambs diets [18,19], although that level of inclusion might be considered high. The Cp, obtained from carob fruit (*Ceratonia siliqua* L.), contains several classes of polyphenols such as gallic acid, catechin, epicatechin, myricetin, quercetin, hydrolysable tannins, and CTs, characterised by their antioxidant activity [20].

Some polyphenols, such as (−)-epicatechin, epigallocatechin, and gallic acid can act as co-antioxidants, by regenerating or protecting Vit E [21]. Gobert et al. [2] revealed that plant extracts rich in polyphenols (non-defined) combined with Vit E (155 IU/kg diet) in a PUFA-rich diet significantly enhanced the α-tocopherol deposition and lowered the lipid oxidation in steaks from cull cows compared to those fed a diet supplemented just with Vit E for a 100 days of the finishing period. In lamb meat, Jerónimo et al. [22] found similar results fattening light lambs for 42 days with a CTs source (20 g of *Cistus ladanifer* L./kg of dry matter —DM—) and low Vit E (22.5 IU/kg of pelletised concentrate). Thus, a complementary mechanism between these compounds was proposed, since Vit E acts as a chain-breaking antioxidant in the lipid fraction, whereas the polyphenols (e.g., catechins) scavenge ROS aqueous phases [2]. We hypothesised that supplementing lamb diets with Cp might evidence a potential synergistic or replacing effect on the Vit E and meat antioxidant capacity and shelf-life. Therefore, this study evaluated the combined effects of dietary carob pulp (0 and 20%) and vitamin E (40 and 300 IU/kg of feed), in fattening lambs for 41 days, on their meat quality and shelf-life stored under MAP.

## 2. Materials and Methods

### 2.1. Animal and Experimental Design

This study was conducted at the experimental facilities and commercial abattoir of BonArea Group (Guissona, Spain). All procedures were supervised and approved by the Ethics Committee for Experimental Animals under protocol number CEEA 02-03/21, procedure N° 01.

A total of 48 commercial crossbred lambs (Romane × Berberine × Ripollesa) of both sexes (half females and half entire males) were collected from a larger group of animals. Initially, seventy-two lambs (13.3 ± 1.3 kg body weight—BW—and 41.8 ± 5.8 days old) were weaned and randomly distributed into 12 group pens, each housing 6 lambs (three females and three males). After a 7-day adaptation period with commercial pelletized concentrate plus barley straw, pens were randomly assigned to one of four treatment groups within a 2 × 2 factorial design, with two levels of Cp (0 or 20%) and two levels of Vit E (40 or 300 IU/kg feed), resulting in four isoenergetic (8.0 MJ net energy/kg of DM) and isoproteic (17.4% crude protein) concentrates: 0%Cp–Low Vit E (Control diet); 0%Cp–High Vit E (Control diet plus 300 IU of Vit E); 20%Cp–Low Vit E (20% of Cp and 40 IU of Vit E); 20%Cp–High Vit E (20% of Cp plus 300 IU Vit E). The specific levels of carob pulp and vitamin E supplementation were chosen based on the results of previous dose–response experiments. These experiments demonstrated that both ingredients, when administered independently as Cp [19] and Vit E [23,24], had a significant impact on various quality attributes of lamb meat.

The Control diet was composed of cereals (20.4% corn, 36.1% barley, 5.01% wheat), soybean co-products (15.0% hulls and 19.8% meal), 0.2% palm oil, and 3.44% minerals and vitamins. The Cp group received a concentrate in which most barley and wheat were replaced with 20% of Cp and 1.85% of palm oil was added to balance diets. In addition, High Vit E groups were supplemented with 0.05% all-rac-α-tocopheryl acetate (CUXAVIT E 50, Kaesler Nutrition, Cuxhaven, Germany, Code 3a700). Concentrates in pelleted form, barley straw, and water were offered *ad libitum* during the 41 days of experimental fattening. The ingredients and chemical composition of diets was detailed elsewhere [25] and in Table S1. Table 1 describes the feed FA profile and secondary compounds.

### 2.2. Feed Analyses

Samples of the experimental diets were collected throughout the study and freeze-dried for subsequent analyses. The content of carotenoids and tocopherol isomers was determined following the methodology described by Bottegal et al. [19], which included an extraction procedure, and the dry residue obtained was injected into an ACQUITY UPLC H-Class liquid chromatograph (Waters, Milford, MA, USA). The total content of polyphenols and CTs (obtained as the sum of extractable CTs, protein-bound CTs, and fibre-bound CTs) were extracted and quantified according to Rufino-Moya et al. [26] using a Heλios β spectrophotometer (Thermo Electron Corporation, Waltham, MA, USA).

The ether extract was determined with an XT10 Ankom extractor (Ankom Technology Corporation, Fairport, NY, USA) according to AOCS (Procedure Am 5-04, [27]). The FA of feedstuffs were extracted following the method described by Sukhija and Palmquist [28] after an optimisation process explained in detail by Baila et al. [29]. The C19:0 was used as internal standard and the concentration was determined using a Bruker Scion 436-GC gas-chromatograph (Bruker, Billerica, MA, USA) with a flame ionisation detector equipped with a CP-8400 autosampler and an SP-2560 capillary column (100 m × 0.25 mm ID × 0.20 μm; Sigma-Aldrich, Saint Louis, MO, USA).

### 2.3. Animal and Meat Sampling

At the end of the fattening period and after 4 h of fasting (with *ad libitum* water access), 72 animals were individually weighed (25.3 ± 1.7 kg of BW) and transported (3 km) to a commercial abattoir. After arrival, the animals were immediately captive bolt stunned and slaughtered according to EU welfare guidelines. In the abattoir, a total of 48 carcasses were randomly selected, 12 per treatment (6 females and 6 males). On the slaughter line, the hot carcass weight (without head) was recorded, and the hot carcass dressing percentage was calculated. At 60 min post-slaughter, the subcutaneous fat colour was measured at the base of the tail using a portable Minolta CM-700d spectrophotometer (Konica Minolta Sensing Inc., Osaka, Japan). This equipment has an 8 mm diameter measurement area, a specular component, and a 0% UV, standard illuminant D65 (colour temperature of 6504 K): observer angle 10° and zero. Carcasses were chilled for 12 h at 4 °C. Later, five 1.5 cm thick chops were collected from the medium zone of each right leg and were randomly assigned to one of five display times (0, 9, 11, 13 or 15 days). The meat samples, except for those stored for 0 days, were packaged in MAP (80% O_2_ and 20% CO_2_). Polyethylene trays containing absorbent pads were used and wrapped in a breathable plastic film (nylon, vinyl alcohol, and polyethylene). All trays were stored in the dark at 4 ± 1 °C for the specified display time. Each tray contained three chops (all from different diet groups) which were individually weighed at the beginning and end of the storage to estimate purge losses during storage.

### 2.4. Intramuscular Fatty Acid Composition, Tocopherol Isomers, and Cholesterol Content 

A total of 15 g of *Semimembranosus* muscle (SM) was taken from 0-day samples and freeze-dried (Freeze-dryer gamma 2–16 LSC-plus, Martin Christ, Osterode am Harz, Germany) for subsequent FA composition, tocopherol, and cholesterol content analyses. The FA of 500 mg of the freeze-dried meat samples were extracted using C23:0 as an internal standard following the methodology previously described by Bottegal et al. [19]. All samples were processed through a two-step methylation procedure to obtain FA methyl esters (FAME). The identification of FA was performed using the gas chromatograph described in the determination of FA in the feed. A total of 1 μL of the sample with 1/50 split was injected (at 250 °C) and FA identification was based on the retention times as compared with those of the standard FAME mixtures: GLC-532, GLC-401, GLC-463, and GLC-538 (Nu-Check Prep, Elysian, USA). The FAME were quantified following the indications of UNE-EN ISO 12966-4:2015 [30]. The highly peroxidizable PUFA (HP-PUFA) indicates the level of FA more susceptible to oxidation and was calculated as the sum of PUFA with ≥3 double bonds [31].

Tocopherol isomers (α-,γ-) and cholesterol content were analysed by liquid chromatography, as described in the Section 2.2, following the methodology by Blanco et al. [32]. Briefly, 200 mg of freeze-dried meat underwent an overnight saponification process with 200 mg of L-ascorbic acid and a saponification solution (3 mL, 10% *w*/*v* KOH in 50:50 ethanol:water v:v). Then, the extraction was conducted with 5 mL of n-hexane:ethyl acetate 9:1 v:v and 5 µg mL^−1^ of the butylated hydroxytoluene mixture. The mixture was vortexed, shaken (600 rpm, 15 min), and centrifuged (2000× *g*, 10 °C, 5 min). The organic upper layer was evaporated at 40 °C for 30 min using a rotary vacuum evaporator. Finally, the residue was dissolved in 1 mL of mobile phase (75:15:10 acetonitrile:methanol:dichloromethane v:v:v) and injected into the liquid chromatograph. 

### 2.5. Meat Colour and Lipid Oxidation

At the end of the target display time, chops were removed from packs and bloomed for 30 min before colour measurements. Meat colour parameters (L*-lightness-, a*-redness-, b*-yellowness-coordinates in the CIELab space) were recorded, by duplicate, on the SM of every chop using the aforementioned portable Minolta CM-700d spectrophotometer. Reflectance spectra from 400 to 700 nm wavelength were recorded to estimate the metmyoglobin formation (MMb, %) as = 100 × {1.395 − [(A572 nm − A700 nm)/(A525 nm − A700 nm)]}. Also, chroma (C* = √(a*^2^ + b*^2^)) and hue angle (h° = 57.29 × tan − 1(b*/a*)) were calculated. The pH was also measured at 24 h *post mortem* and after 15 days of display time, using a two-point calibration (pH 4.00 and pH 7.00) pH metre (G-PH7V, XS instruments, Carpi, Italy) equipped with a penetration electrode in the meat samples.

After colour measurements, 10 g of SM were vacuum-packed and frozen (−80 °C) until the muscle lipid oxidation analysis was performed through the thiobarbituric acid-reactive substance technique (TBARS). The TBARS values were expressed as mg malondialdehyde (MDA)/kg of meat. The lean meat sample was mixed with 20 mL of 100 g L^−1^ trichloroacetic acid and 50 µL of 7.2% (*w*/*v*) butylated hydroxytoluene in ethanol using a Miccra D-8 Homogenizer (Falc Instruments, Treviglio, Italy). The homogenate was then centrifuged (4000 rpm, 15 min and 4 °C) and the supernatant was filtered. A total of 1 mL of the filtered extract was vortexed with 10 mM 2-thiobarbituric acid and later incubated (100 °C, 45 min and 100 rpm) to form MDA-TBA2. After cooling, 150 µL was pipetted into a 2 mL amber screw-cap vial with 850 µL of a mixture of ACN:ultrapure water at a ratio of 30:70 (v:v). The final extracts were injected into the liquid chromatograph.

### 2.6. Microbiological Count 

Microbiological analyses were conducted on *Bicep femoris* muscle samples from three lambs per dietary treatment at 24 h *post mortem* and at 15 days of display. Once opening the tray, 10 g of meat was aseptically extracted within a laminar flow cabinet and later homogenised with 90 g of saline peptone water for 1 min in a sterile plastic bag equipped with a filter, using a Stomacher Masticator (IUL, S.A., Barcelona, Spain) at room temperature. Subsequently, serial decimal dilutions were prepared in sterile peptone water and, in duplicate, 1 mL or 0.1 mL samples of appropriate dilutions were spread on plate count agar (Oxoid, Ireland). Incubation of the plates was carried out at 30 ± 1 °C for 72 h and at 6.5 ± 1 °C for 10 days for counting aerobic mesophilic and psychrotrophic microorganisms, respectively. Those counts were performed according to the ISO 4833-1:2013 [33] and ISO 17410:2019 [34], respectively. 

### 2.7. Statistical Analysis

All statistical analyses were performed using the Infostat software 2020 (Centro de Transferencia InfoStat, Universidad Nacional de Córdoba, Córdoba, Argentina). The carcass traits and FA content, tocopherol and cholesterol content, and microbial count in the meat were analysed through analysis of variance with a general linear model. The model used was the following:

y_ijk_ = µ + α_i_ + β_j_ + (α × β)_ij_ + S_k_ + ε_ijkl_,

where y_ijk_ = dependent variable, µ = overall mean, α_i_ = carob inclusion level effect (i = 0%, 20%), β_j_ = vitamin E effect (j = low, high), (α × β)_ij_ = interaction between carob pulp and vitamin E, S_k_ = sex effect (k = female, male) and ε_ijkl_ = residual error. Singles interactions between sex and other factors were tested but no significant effects were detected, thus those interactions were removed from the model.

Microbial counts were log-transformed (log_10_) before running the statistical model to approximate the normality of data. 

A mixed linear model for repeated measures was used to analyse the meat colour attributes evolution, storage purge losses, and lipid oxidation considering Cp, Vit E, sex, days of display, and the single interactions as fixed factors, whereas the individual animal nested within the pen was included as a random factor:

y_ijkmn_ = µ + α_i_ + β_j_ + (α × β)_ij_ + T_k_ +(α × T)_ik_ + (β × T)_jk_ + S_m_ + L_n_(p) + ε_ijkmnl_

where y_ijkmn_ = dependent variable, µ = overall mean, α_i_ = fixed effect of carob inclusion level (i = 0%, 20%), β_j_ = fixed effect of vitamin E (j = low, high), (α × β)_ij_ = fixed effect of interaction between carob pulp and vitamin E, T_k_ = fixed effect of display time (k = 0, 9, 11, 13, 15), (α × T)_ik_ = fixed effect of interaction between carob pulp inclusion and display time, (β × T)_jk_ = fixed effect of interaction between vitamin E supplementation and display time, Sm = fixed effect of sex (m = female, male), Ln(p) = the random effect of the individual nested within the pen, and εijkmnl = residual error. To model the experimental error, various variance–covariance matrices were tested. The matrix with the lowest Akaike Information Criterion (AIC) and Bayesian Information Criterion (BIC) was selected as the final error structure, which was determined to be a first-order autoregressive AR(1) model.

Significance was declared at *p* ≤ 0.05. When interactions showed significant differences between means, Tukey’s multiple-comparison test was performed. Interactions that were not significant were excluded from the model.

## 3. Results

### 3.1. Carcass Traits and Caudal Fat Colour

There were no significant interactions (*p* > 0.05) between Cp and Vit E, nor between Cp or Vit E and sex in the carcass parameters; thus, the main effects on carcass traits are presented separately in Table 2. 

The Cp inclusion did not affect (*p* > 0.05) the carcass attributes or caudal fat colour. In addition, carcass traits were not affected (*p* > 0.05) by the Vit E doses, but it did modify caudal fat colour. Therefore, in the High Vit E group, the caudal fat lightness (L*) was lower (*p* < 0.05) whereas redness (a*) and yellowness (b*) were higher (*p* < 0.05) compared to the Low Vit E group. Regarding sex, females showed lower final BW (*p* < 0.001) and carcass weight (*p* < 0.01) but higher dressing percentage (*p* < 0.05) than male lambs. However, fat carcass colour was not affected (*p* > 0.05) by sex.

### 3.2. Lipid Composition and Antioxidants in Meat

The interactions between Cp, Vit E and sex were tested but no effect (*p* > 0.05) was found on the FA profile, or α-tocopherol and cholesterol content in meat, hence the main effects are shown separately (Table 3). The total FA content was not affected by any factor studied (*p* > 0.05). The FA data are expressed as g of FA/100 g of total FA, since the sum of FAME was similar between groups. 

The FA composition was slightly affected by Cp inclusion, as only the sum of odd and branched-chain (OBCFA) was reduced by Cp (*p* < 0.05). This is due to the lower *anteiso*-BCFA content (*p* < 0.001) observed in meat from the 20% Cp-group compared to lambs from the 0% Cp-group. Vitamin E did not affect the content of any FA or the sum of saturated FA (SFA), monounsaturated FA (MUFA), or PUFA (*p* > 0.05). Surprisingly, cholesterol content was greater in the High Vit E group compared to the Low Vit E group (*p* < 0.001).

Sex revealed effects on minor FA; female lambs showed a higher concentration of the sum of SFA methyl-acetals, *anteiso*-BCFA, and total OBCFA than males in meat (*p* < 0.05). Likewise, the sum of SFA, MUFA, PUFA, or the n-3/n-6 ratio were unaffected by the sex (*p* > 0.05).

Concerning the analysed antioxidants, the α-tocopherol isomer was affected by both Cp and Vit E supplementation (*p* < 0.05), whereas the γ-tocopherol and retinol content in meat were similar across diets (*p* > 0.05). Sex did not affect any of these variables (*p* > 0.05). The dietary inclusion of Cp reduced the α-tocopherol content in meat (*p* < 0.05). However, the High Vit E level increased the α-tocopherol content in meat (*p* < 0.001) compared to Low Vit E. 

### 3.3. Meat Colour Evolution and Lipid Oxidation Evolution

**Figure 1:**
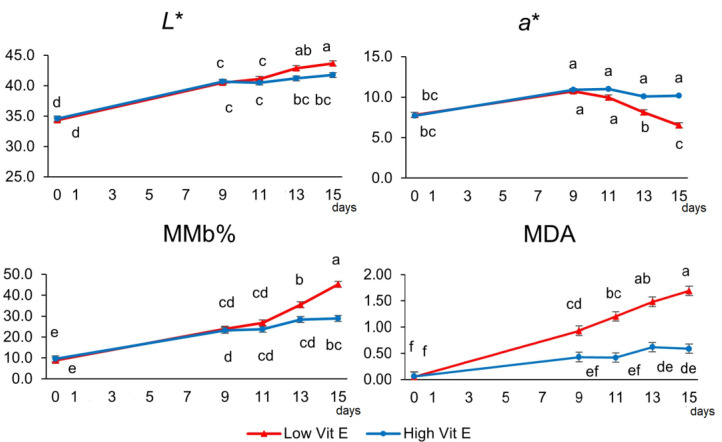
Effect of vitamin E supplementation (40 vs. 300 IU of Vit E/kg, Low and High, respectively) on lightness (L*), redness (a*), calculated metmyoglobin percentage (MMb), and malonaldehyde (MDA, expressed as mg/kg of meat) concentration in meat stored in MAP (80% O_2_ + 20% CO_2_) up to 15 days. Different letters indicate significant differences (*p* < 0.05) between Vit E groups and storage days.

No interactions between factors (Cp, sex and day of storage) were detected (*p* > 0.05) in the evolution of the variables studied during display time. The meat colour attributes and purge losses were unaffected by Cp inclusion during the 15 days of MAP storage (Table 4, *p* > 0.05). Likewise, an interaction effect (*p* < 0.01) was found between the Vit E and display time in almost all variables (Figure 1). The L* parameter increased linearly up to 15 days in the Low Vit E group, meanwhile in the High Vit E group, L* increased up to 9 days and later remained stable. A similar pattern was observed on the hue angle. Regarding a* and chroma, it increased up to 9 days and remained stable until day 15 in the High Vit E group; however, in the Low Vit E group, a* showed the nadir value on day 15. A linear increase was observed in MMb during storage (*p* < 0.001); meanwhile, the Low Vit E group showed higher MMb values from day 13 onwards compared with the High Vit E group. The Cp did not affect MDA production (*p* > 0.05). In addition, lipid oxidation was greater in the Low Vit E group, as its MDA concentration was higher than in the High Vit E group from day 9 onwards (*p* < 0.05). 

The purge losses showed an interaction between Vit E and display time (*p* = 0.043). Differences between Vit E groups were observed exclusively on day 15, when Low Vit E reached a greater value of purge loss than High Vit E (3.28 vs. 2.57 ± 0.17%, *p* < 0.05).

**Table 4:** Effect of dietary inclusion of carob pulp (0 vs. 20% Cp) and vitamin E (40 vs. 300 IU Vit E/kg feed) and sex (female and male) on meat colour evolution and lipid oxidation (MDA) of *Semimembranosus* muscle stored in MAP up to 15 days in darkness.

	Cp ^1^(%)		Vit E ^2^		Day					Sex			*p*-Value			
	0	20	Low	High	0	9	11	13	15	Female	Male	SEM ^3^	Cp	Vit E	Day	Sex
Lightness (L*)	39.9	40.3	40.4	39.8	34.4 ^c^	40.6 ^b^	40.8 ^b^	42.0 ^a^	42.7 ^a^	39.9	40.3	0.30	0.400	0.059	<0.001	0.252
Redness (a*)	9.24	9.34	8.62	9.95	7.75 ^c^	10.8 ^a^	10.5 ^a^	9.08 ^b^	8.33 ^c^	9.4	9.2	0.23	0.715	<0.001	<0.001	0.507
Yellowness (b*)	14.2	14.3	14.3	14.2	9.81 ^c^	15.0 ^b^	15.4 ^ab^	15.5 ^a^	15.6 ^a^	14.4	14.1	0.13	0.428	0.407	<0.001	0.167
Chroma (C*)	16.9	17.1	16.7	17.4	12.5 ^d^	18.4 ^ab^	18.5 ^a^	17.9 ^bc^	17.7 ^c^	17.3	16.7	0.17	0.412	0.007	<0.001	0.032
Hue angle (h°)	56.7	56.6	58.5	54.9	51.6 ^d^	54.0 ^cd^	55.8 ^c^	59.7 ^b^	62.2 ^a^	56.9	56.4	0.65	0.926	<0.001	<0.001	0.527
MMb ^4^, %	25.8	25.0	28.0	22.8	9.16 ^d^	23.5 ^c^	25.3 ^c^	31.9 ^b^	37.1 ^a^	26.2	24.6	1.0	0.515	<0.001	<0.001	0.180
MDA ^5^, mg/kg meat	0.74	0.75	1.07	0.42	0.05 ^c^	0.68 ^b^	0.81 ^b^	1.05 ^a^	1.14 ^a^	0.8	0.69	0.06	0.857	<0.001	<0.001	0.178
Storage purge loss, %	2.40	2.61	2.67	2.33	−	2.04 ^c^	2.53 ^b^	2.54 ^b^	2.9 ^a^	2.61	2.40	0.14	0.288	0.101	<0.001	0.127

^1^ Carob pulp, ^2^ Vitamin E, ^3^ standard error of means, ^4^ metmyoglobin formation, ^5^ malondialdehyde. ^a,b,c,d^ Different letters in the same row indicate differences (*p* < 0.05) between display days. Significant interaction was detected between Vit E and display time on L*, a*, MMb, and MDA: see Figure 1.

### 3.4. Microbial Spoilage Under MAP Storage

The evaluation of the shelf-life of meat based on microbial counts showed a similar initial mesophilic bacterial load in all groups (*p* > 0.05, Table 5), which is important because it will define the meat shelf-life under MAP. The pH was not affected by diets (*p* > 0.05).

**Figure 2:**
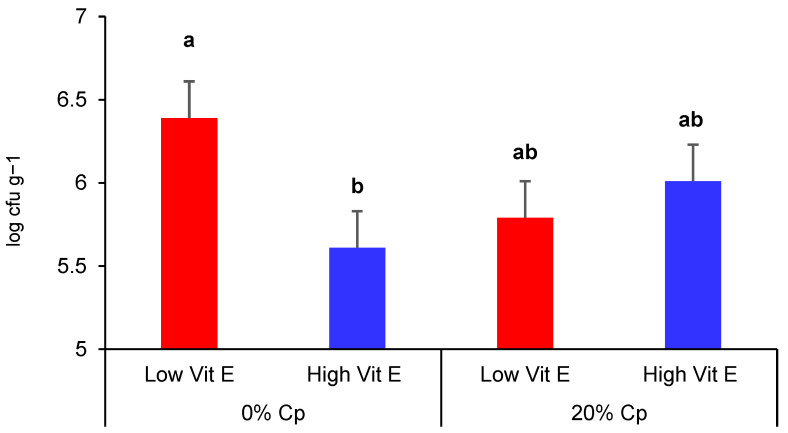
Interaction between carob pulp (0 vs. 20% Cp) and vitamin E doses (40 vs. 300 IU/kg, Low and High, respectively) on the total psychrotrophic count (expressed as log CFU/g of meat) in meat stored 15 days. Different letters indicate differences (*p* < 0.05) between groups.

Regarding the total psychrotrophic count on meat, it was similar across diets at day 0 (*p* > 0.05) but it was affected by the interaction (*p* < 0.05) between Cp and Vit E on day 15 of storage (Figure 2). The 0% Cp–High Vit E diet reduced (*p* < 0.05) the total psychrotrophic count in meat stored for 15 days compared to 0%Cp–Low Vit E, whereas the Cp diets produced intermediate effects either combined with low or high doses of Vit E. 

## 4. Discussion 

Lamb carcass parameters were not affected by Cp inclusion, which is in agreement with other studies including up to 30–35% of Cp in lamb diets [18,35]. These findings suggest that replacing cereals, such as barley, with 20% of Cp in lambs’ diets does not impair carcass traits as long as diets were properly balanced in nutrients and net energy. Also, Vit E did not affect carcass traits, which is consistent with previous studies [36,37] where lambs supplemented with different Vit E doses (30–500 IU/kg) showed no effects on those variables. If the minimum requirement of Vit E (20–40 IU of Vit E/kg feed) for healthy lambs is met, the impacts on carcass traits may be negligible [38]. 

A study conducted in the same facilities and crossbred line of lambs found that males presented a greater average daily gain than females but had similar carcass traits [18]. Facciolongo et al. [39] reported productive differences between sexes and observed a higher carcass dressing in females than males, linked to greater adipose content in females. The lower carcass dressing obtained in males can be partially attributed to the weight of the testes and the higher central or intra-abdominal accumulation of fat which contributes to a greater proportion of non-carcass components [40].

Caudal fat colour was only affected by Vit E. The lipophilic condition of the Vit E may be related to the yellowing of the fat carcass. Few articles have reported the effects of Vit E supplementation on the subcutaneous caudal fat colour. For example, Ripoll et al. [37] found no differences on this variable when supplementing High Vit E in the concentrate. Interestingly, Cp is rich in carotenoids, including β-carotene, lycopene, and zeaxanthin [41]. It explains the elevated total carotenoid content observed in the Cp diets, which in turn may be linked to the increased yellowness reported in Cp concentrates [18]. However, the higher carotenoid content in the Cp diets was insufficient to generate differences in carcass fat yellowing. Also, Priolo et al. [42] found no difference in the fat colour of light lambs fed between 0 and 20% Cp.

The Cp inclusion did not modify the total FA content of lamb meat. Similarly, no differences were detected in the content of intramuscular fat by Gravador et al. [43], who fed lambs with three doses of Cp (0, 24 and 35%). In the current study, some changes were detected in the FA profile of meat due to the dietary inclusion of 20% of Cp, such as a reduction in the BCFA content. These observations agree with the results reported by Bottegal et al. [19], feeding lambs with a 30% Cp diet compared to the 0% Cp diet. The study of BCFA reflects both the abundance and the activity of microbial groups, standing as a useful tool to monitor the feeding regime (e.g., starch or fibre) [44]. Indeed, the ruminal bacteria classified as cellulolytic contain high amounts of *iso*- and *anteiso*-FA compared with amylolytic bacteria. Vasta et al. [45] evaluated the impact of tannins extracted from Cp *in vitro* ruminal fermentation and reported a reduction in the production of *iso*-, *anteiso*-, and *odd*-chain FA compared with the control. The mentioned reduction was related to a lower activity of ruminal microorganisms, especially of cellulolytic bacteria. Natalello et al. [46], feeding lambs with pomegranate by-products rich in flavonoids, anthocyanidins, and tannins (mostly represented by ellagitannins), found a decrease in the BCFA content in meat. Likewise, Baila et al. [29] demonstrated that feeding ewes with sainfoin, a CTs-rich forage, can also reduce the BCFA content in milk. Additionally, the lower BCFA content in meat could also be due to the lower starch content in Cp. In line with this, it was revealed that lower ruminal propionate production, occurring in sheep fed diets with low dietary starch content, leads to a lower availability of propionate to peripheral tissues, which would mean a lower production of methylmalonyl-CoA for OBCFA synthesis in the subcutaneous fat [47].

The lack of effect of Vit E on the FA profile aligns with findings from other studies where lambs received Vit E supplementation ranging from 45 to 551 IU/kg of feed [48]. Although high doses of Vit E have been suggested to affect the FA profile by potentially altering ruminal biohydrogenation or limiting PUFA oxidation [38,49,50], our results did not indicate such effects. Specifically, the *trans*10/*trans*11-C18:1 ratio is commonly used as an indicator of changes in the ruminal biohydrogenation of unsaturated FA and bacterial populations [48], but it was unaffected by High Vit E. The protective effect of Vit E on PUFA has been studied extensively; however, in the current study, no differences in PUFA content were observed between the groups. This was likely because the FA profile was assessed in samples from day 0, when oxidative stress was minimal.

Meat cholesterol content increased when high doses of Vit E were supplemented in the diets. In line with this, the supplementation of lambs with 500 mg of Vit E/kg of feed up-regulated the expression of some genes related to the biosynthesis of cholesterol, sterol, and steroid in the subcutaneous fat, compared to a control group [51]. However, in the literature, the effects of Vit E on cholesterol metabolism and meat deposition are inconclusive and remain to be determined. For instance, neither Salvatori et al. [52] nor Vincenti et al. [53] observed effects of supplementing Vit E on the cholesterol content of lamb meat or beef, respectively. Other studies showed that supplementation of linseed plus Vit E can reduce the cholesterol deposition in beef. Despite measuring cholesterol on day 0, the potential of Vit E to quench free radicals and consequently protect phospholipids and cholesterol from oxidation [3] may have led to increasing its content in this study. 

As mentioned, the similar carcass parameters and total FA content between Cp or Vit E groups indicate that diets were correctly balanced to meet the nutritional requirements of light lambs. This is also supported by the lack of effect on plasmatic lipid compounds in the lambs between groups [25].

In Spanish light lambs, sex is not expected to modify the FA profile, since, as Horcada et al. [54] concluded, the impact of sex in the meat FA profile is not as important as the diet. In this study, minor changes in the FA profiles were related to sex. In fact, those minor FA affected by sex (SFA Me and BCFA) are mainly prone to be influenced by changes in the ruminal microbial populations due to variations in feeding patterns.

The reduction in meat α-tocopherol content due to the dietary Cp inclusion was also observed by Bottegal et al. [19], who found a similar decrease in the α-tocopherol content in lambs fed with 30% Cp (supplemented with 300 IU of Vit E/kg of feed) compared with lambs fed diets with 0 or 15% Cp. However, other studies have shown a protective and regenerative effect of Vit E when combined with other polyphenol sources [13], potentially increasing α-tocopherol content in meat [22,55]. In the case of Cp, the effect on α-tocopherol may differ due to its high proportion of protein-bound CTs [25] and may limit its antioxidant activity compared to other sources rich in extractable CTs which are not bound to protein of fibre. This could explain the contrasting results observed in our study regarding α-tocopherol levels. 

The supplementation of High Vit E was expected to increase the α-tocopherol content in meat, since a positive linear relationship between the level and length of Vit E supplementation and the α-tocopherol deposited in lamb meat has been widely documented [23,37]. In the current trial, supplementing lambs with 300 IU of Vit E/kg for 41 days increased 2.16-fold the α-tocopherol SM content in comparison to the Low Vit E group (40 IU).

Most studies supplementing Cp in lambs’ diets (15–24%) observed scarce or null effects on meat colour [19,42,43]. Meat colour is one of the most determining aspects that consumers consider when making their purchasing decisions [56]. Therefore, maintaining or preventing meat discolouration during display becomes crucial when incorporating agricultural by-products into animal diets.

Phenolic acids and several flavonoids as well as CTs are considered powerful antioxidants which control lipid oxidation during meat storage [57]. Interestingly, in the current study, Cp did not apparently reduce MDA evolution, while the α-tocopherol content in the 20%Cp group was lower than in the 0%Cp group. These results might suggest that Cp inhibited lipid oxidation in some way, as MDA levels did not increase under pro-oxidant conditions in the Cp group. Indeed, higher doses of Cp (30–35%) in lamb diets did not control lipid oxidation [19,43]. However, it is worth noting that these studies elevated the content of PUFA in meat when lambs were fed Cp diets compared to control diets. Therefore, the authors speculated that Cp polyphenols (mostly CTs) minimised oxidation under prooxidative conditions (i.e., high PUFA deposition) compared to animals fed a control diet. In addition, polyphenols, even CTs, present a great diversity of chemical structures, defining the way to inhibit oxidative reactions. Accordingly, Wu et al. [58] suggested that the antioxidant mechanisms of flavonoids (keampferol, myricetin, and quercetin) go beyond the strong hydrogen-donating and transition metal-chelating abilities, including complex interactions with hemo-proteins.

Vit E exerted a preservative effect on meat colour, since the High Vit E group improved a* values and controlled the MMb formation, being always below 30%. The a* parameter is directly linked to the reddish appearance of meat and is positively related to Vit E supplementation [59]. Although the high oxygen atmosphere favours redness because it promotes the presence of oxymyoglobin (lower MMb), low doses of Vit E could not preserve stable redness for more than 11 days. To detect meat discolouration, 30% MMb is considered a threshold. This colour preservative effect was likely related to the control carried out on lipid oxidation. Controlling lipid oxidation is essential because it leads to deterioration in meat colour, texture, nutritional value, and flavour [59]. The antioxidant properties of Vit E have been widely researched [60] and this study confirmed that the inclusion of 300 IU of Vit E/kg for 41 days in lambs diets may control lipid oxidation in meat storage under MAP in darkness until 15 days. Additionally, the content of α-tocopherol in SM in the High Vit E group was 2.66 mg α-tocopherol/kg of meat, which overcame the threshold of 1.9–2.3 mg/kg which may be considered optimal to avoid an increase in lipid oxidation before 11 days of MAP storage [19]. 

It is worth noting that High Vit E reduced the purge losses, especially after 15 days of storage, in comparison with the Low Vit E group. In this sense, it was suggested that Vit E in ruminants’ diets improves the water-holding capacity of meat, since this maintains the integrity of the muscle cell membrane and prevents the passage of muscle slurry through the cell membrane [61].

Finally, at day 15 of storage, the total mesophile count was below the limit considered unsuitable for consumption (7 log CFU g^−1^ meat [62]); it is likely that, the MAP conditions (containing 20% CO_2_) helped to control microbial growth [5]. Supplementation with High Vit E reduced the total psychrotrophic count compared to the Low Vit E group, while Cp showed intermediate values. Similarly, Guerra-Rivas et al. [63] observed that feeding lambs with 500 IU Vit E/kg controlled the total viable counts in meat stored up to 14 days in MAP compared to the group fed grape pomace (a tannins source). These findings seem to indicate that Vit E was effective in preventing the growth of psychrotrophic microorganisms and that Cp may counterbalance the effects of low Vit E. The antimicrobial effect of this vitamin was documented previously in suckling and fattening lambs, but further explanation was not elucidated [62,63]. Contradictorily, other studies [60,64] have shown that high doses of Vit E (250–1000 IU Vit E) do not contribute to controlling microbial growth in MAP, although Vit E did increase lipid and colour stability. Overall, research supporting the antimicrobial effect of Vit E is quite limited [38]. 

On the other hand, the antibacterial effects of hydrolysable and condensed tannins have been reported on the total mesophilic and psychrotrophic bacteria count in lamb meat storage up to 7 days [65]. More specifically, some studies have reported that flavanones, quercetin, and catechins from carob are responsible for antimicrobial properties tested in vitro [66,67]. However, in the current work, the dietary Cp inclusion did not show a clear antimicrobial effect in lamb meat, likely due to the low muscle deposition of secondary compounds. Additionally, protective effects against microbial spoilage would be expected if carob extracts were applied directly to meat products [68].

## 5. Conclusions

The synergistic relationship effects between Cp and Vit E on the α-tocopherol content and antioxidant capacity of lamb meat are not evident. It is feasible to include 20% of carob pulp in the diets of fattening lambs without impairing the quality or shelf-life of the meat and only minor changes in the fatty acid meat profile are produced, which might be linked to alterations in the ruminal microbiota. Despite the reduction in the α-tocopherol content of the meat resulting from dietary carob pulp, there is no increase in lipid oxidation. Supplementing 300 IU of vitamin E/kg of feed in fattening lambs’ diets would be recommended, as it promotes its muscle deposition (>2.5 mg/kg of meat) and exerts protective effects against lipid oxidation and discolouration in meat stored up to 15 days in modified atmosphere packs without affecting the fatty acid profile. Despite the lambs’ young age, impacts on carcass traits are observed between sexes with null or minor effects on meat quality.

## Figures and Tables

**Table 1:** Means ± standard deviation fatty acids (FA) composition and secondary compounds of the diets used. Control diet and carob pulp diets contained 0 and 20% of carob pulp, respectively.

Item	Control Diet	Carob Pulp Diet
Lutein, µg/g DM	1.51 ± 0.05	2.98 ± 1.14
β-carotene, µg/g DM	1.52 ± 0.07	9.23 ± 0.83
Total carotenoids ^1^, µg/g DM	4.70 ± 0.09	14.3 ± 1.07
α-tocopherol, mg/kg DM		
Low ^2^	37.3	40.0
High	241	283
γ-tocopherol, mg/kg DM		
Low	11.6	12.9
High	11.7	14.4
Total tocopherols, mg/kg DM		
Low	49	53
High	253	297
Total polyphenols, g tannic acid eq./kg DM	4.34 ± 0.10	6.46 ± 0.63
Total condensed tannins ^3^, g carob pulp total CTs-eq./kg DM	1.45 ± 0.47	18.9 ± 4.36
Ether extract (%)	2.56 ± 0.01	3.36 ± 0.01
Fatty acid profile (g/100 g of total FA)		
C12:0	0.13 ± 0.01	0.21 ± 0.02
C14:0	0.36 ± 0.01	0.60 ± 0.01
C16:0	25.2 ± 0.33	31.2 ± 0.42
C17:0	0.30 ± 0.05	0.23 ± 0.07
C18:0	9.16 ± 0.07	7.42 ± 0.65
*cis*9-C16:1	0.11 ± 0.01	0.11 ± 0.01
*cis*9-C18:1	18.8 ± 0.22	27.5 ± 0.59
*cis*11-C18:1	0.05 ± 0.03	0.06 ± 0.03
C20:1	0.04 ± 0.01	0.03 ± 0.01
C18:2-n6	42.8 ± 0.45	31.0 ± 0.52
C18:3-n3	3.00 ± 0.16	1.60 ± 0.10
Sum of SFA	35.2 ± 0.31	39.7 ± 1.03
Sum of MUFA	19.0 ± 0.23	27.7 ± 0.60
Sum of PUFA	45.8 ± 0.45	32.6 ± 0.58

DM—dry matter, SFA—saturated fatty acids, MUFA—monounsaturated fatty acids, PUFA—polyunsaturated fatty acids. ^1^ Represent the sum of zeaxanthin, lutein, and β-carotene content. ^2^ Low and High correspond to the diets with 40 and 300 IU Vit E/kg, considering 1 mg of all-rac-α-tocopheryl acetate synonym of 1 IU. ^3^ Expressed in g of the equivalent of condensed tannins (CTs) purified from carob pulp/kg of DM of feed.

**Table 2:** Effect of dietary inclusion of carob pulp (0 vs. 20% Cp) and vitamin E (40 vs. 300 IU Vit E/kg) in the final body weight, carcass traits and colour of caudal fat of female and male light lambs.

Item	Cp (%) ^1^		Vit E ^2^		Sex			*p*-Value ^4^		
	0	20	Low	High	Female	Male	SEM ^3^	Cp	Vit E	Sex
*n*	24	24	24	24	24	24				
Final body weight, kg	25.4	25.2	25.2	25.4	24.3	26.3	0.30	0.708	0.568	<0.001
Hot carcass weight, kg	11.8	11.9	11.8	11.9	11.5	12.2	0.17	0.600	0.507	<0.01
Carcass dressing, %	46.6	47.4	46.9	47.1	47.5	46.4	0.37	0.129	0.730	0.042
Caudal fat colour										
Lightness (L*)	71.3	72.4	72.8	70.9	71.9	71.9	0.53	0.134	0.016	0.997
Redness (a*)	0.86	1.02	0.56	1.32	0.99	0.89	0.16	0.504	<0.01	0.666
Yellowness (b*)	7.99	7.92	7.37	8.54	7.90	8.00	0.34	0.888	0.020	0.838

^1^ Carob pulp, ^2^ Vitamin E, ^3^ standard error of the means. ^4^ No significant interactions were detected (*p* > 0.05).

**Table 3:** Effect of carob pulp (0 vs. 20% Cp), vitamin E (40 vs. 300 IU Vit E/kg), and sex on the total fatty acids (FA) content, major classes of FA (weight % of total FA methyl esters), cholesterol, and antioxidants content.

	Cp (%)		Vitamin E		Sex		SEM ^1^	*p*-Value ^2^		
Item	0	20	Low	High	Female	Male		Cp	Vit E	Sex
Total FA, mg/g meat	1417	1390	1403	1405	1424	1383	61.3	0.761	0.981	0.640
Fatty acids, g FA/100 g FAMEs										
∑SFA Me	0.88	0.87	0.87	0.88	0.9	0.85	0.020	0.656	0.710	0.043
∑DMA	5.24	5.31	5.4	5.15	5.33	5.23	0.180	0.781	0.352	0.702
∑*iso*-BCFA	1.20	1.12	1.14	1.17	1.22	1.10	0.041	0.207	0.599	0.052
∑*anteiso*-BCFA	0.74	0.68	0.72	0.70	0.74	0.68	0.014	0.001	0.488	0.004
∑OBCFA	1.94	1.80	1.86	1.88	1.96	1.78	0.04	0.013	0.762	0.003
∑SFA	45.6	45.7	45.9	45.5	45.6	45.8	0.24	0.772	0.253	0.461
∑*cis-*MUFA	30.2	31.3	30.2	31.3	31.2	30.3	0.57	0.166	0.193	0.254
∑t*rans*-MUFA	3.18	3.22	3.14	3.26	3.07	3.33	0.180	0.891	0.653	0.325
∑MUFA	33.4	34.5	33.4	34.5	34.3	33.6	0.55	0.143	0.139	0.397
∑CLA	0.29	0.28	0.29	0.29	0.29	0.28	0.010	0.782	0.935	0.800
∑PUFA	21.0	19.7	20.8	20.0	20.2	20.6	0.61	0.146	0.362	0.627
∑PUFA *n* − 6	17.8	16.5	17.6	16.7	16.9	17.4	0.56	0.105	0.302	0.602
∑PUFA *n* − 3	2.06	2.03	1.99	2.09	2.05	2.03	0.070	0.783	0.386	0.804
*n* − 6/*n* − 3	8.80	8.30	8.90	8.19	8.40	8.70	0.300	0.254	0.105	0.492
*trans*10/*trans*11-C18:1	1.16	1.35	1.31	1.2	1.2	1.31	0.07	0.060	0.264	0.280
Rumenic Acid (*cis*9, *trans*11-C18:2)	0.13	0.12	0.12	0.12	0.12	0.12	0.01	0.648	0.943	0.724
PUFA/SFA	0.46	0.43	0.45	0.44	0.44	0.45	0.01	0.164	0.49	0.725
HP-PUFA	8.47	8.35	8.53	8.30	8.34	8.49	0.28	0.769	0.582	0.725
Cholesterol, mg/g meat	0.78	0.75	0.73	0.8	0.76	0.77	0.01	0.090	<0.001	0.594
Fat-soluble antioxidant vitamins, mg/kg meat										
α-tocopherol	2.05	1.84	1.22	2.66	1.86	2.02	0.07	0.036	<0.001	0.120
γ-tocopherol	0.10	0.13	0.13	0.09	0.12	0.11	0.02	0.395	0.163	0.658
Retinol	0.03	0.03	0.03	0.03	0.03	0.03	0.001	0.703	0.232	0.396

FA—fatty acids; SFA—saturated FA; ∑SFA Me—um of saturated methyl FA; ∑DMA—sum of dimethyl-acetals FA; BCFA—branched chain FA; ∑OBCFA—sum of *odd*-, *anteiso*-, and *iso*-BCFA; MUFA—monounsaturated FA; ∑CLA—sum of conjugated linoleic acids; PUFA—polyunsaturated FA; HP-PUFA—highly hydrolysable PUFA (calculated as the sum of PUFA ≥ 3 double bonds). ^1^ Standard error of means. ^2^ No significant interactions between factors were detected (*p* > 0.05).

**Table 5:** Effect of inclusion of carob pulp (0 vs. 20% Cp) or Vit E (40 vs. 300 IU/kg) in lamb diets and display time on pH and microbial count (expressed as log CFU g^−1^) on *Bicep femoris* muscle of male lambs during refrigerated storage at 4 °C.

		Cp (%)		Vitamin E			*p*-Value ^3^	
Item	Day ^1^	0	20	Low	High	SEM ^2^	Cp	Vit E
pH	0	5.73	5.75	5.73	5.75	0.02	0.462	0.368
	15	5.57	5.58	5.56	5.59	0.01	0.961	0.085
Total mesophilic count	0	3.73	3.63	3.68	3.67	0.19	0.716	0.981
	15	5.67	5.54	5.80	5.41	0.24	0.445	0.592
Total psychrotrophic count	0	3.42	3.19	3.37	3.22	0.19	0.739	0.282
	15	6.04	5.9	6.13	5.81	0.16	0.570	0.205

^1^ Days storage under MAP conditions, day 0 corresponds to evaluations performed 24 h *post mortem*. ^2^ Standard error of means. ^3^ Interaction between Cp and Vit E was found (*p* < 0.05) in the total psychrotrophic count in meat stored for 15 days (Figure 2).

## Data Availability

Data presented in this study are available upon request from the corresponding author.

## Supplementary Materials

The following supporting information can be downloaded at: https://www.mdpi.com/article/10.3390/ani14243629/s1, Table S1. Ingredients and chemical composition of experimental diets, with two levels of carob pulp (Cp, 0 vs. 20%) and two doses of vitamin E (Vit E, 40 vs. 300 IU/kg).

## References

[1] Karabagias I., Badeka A., Kontominas M.G. (2011). Shelf Life Extension of Lamb Meat Using Thyme or Oregano Essential Oils and Modified Atmosphere Packaging. Meat Sci..

[2] Gobert M., Gruffat D., Habeanu M., Parafita E., Bauchart D., Durand D. (2010). Plant Extracts Combined with Vitamin E in PUFA-Rich Diets of Cull Cows Protect Processed Beef against Lipid Oxidation. Meat Sci..

[3] Descalzo A.M., Sancho A.M. (2008). A Review of Natural Antioxidants and Their Effects on Oxidative Status, Odor and Quality of Fresh Beef Produced in Argentina. Meat Sci..

[4] Rubio B., Vieira C., Martínez B. (2016). Effect of Post Mortem Temperatures and Modified Atmospheres Packaging on Shelf Life of Suckling Lamb Meat. LWT Food Sci. Technol..

[5] Kowalczyk M., Domaradzki P., Ziomek M., Ska P., Chmielowiec-korzeniowska A., Nuvoloni R., Florek M. (2024). Effect of VP, MAP and Combined Packaging Systems on the Physicochemical Properties and Microbiological Status of Veal from Unweaned Calves. Meat Sci..

[6] Cunha L.C.M., Monteiro M.L.G., Lorenzo J.M., Munekata P.E.S., Muchenje V., de Carvalho F.A.L., Conte-Junior C.A. (2018). Natural Antioxidants in Processing and Storage Stability of Sheep and Goat Meat Products. Food Res. Int..

[7] Landete J.M. (2013). Dietary Intake of Natural Antioxidants: Vitamins and Polyphenols. Crit. Rev. Food Sci. Nutr..

[8] Pateiro M., Barba F.J., Domínguez R., Sant’Ana A.S., Mousavi Khaneghah A., Gavahian M., Gómez B., Lorenzo J.M. (2018). Essential Oils as Natural Additives to Prevent Oxidation Reactions in Meat and Meat Products: A Review. Food Res. Int..

[9] FEDNA (2008). Necesidades Nutricionales Para Rumiantes En Cebo: Normas FEDNA.

[10] Jose C.G., Jacob R.H., Pethick D.W., Gardner G.E. (2016). Short Term Supplementation Rates to Optimise Vitamin E Concentration for Retail Colour Stability of Australian Lamb Meat. Meat Sci..

[11] Ripoll G., Joy M., Muñoz F. (2011). Use of Dietary Vitamin E and Selenium (Se) to Increase the Shelf Life of Modified Atmosphere Packaged Light Lamb Meat. Meat Sci..

[12] Álvarez-Rodríguez J., Urrutia O., Lobón S., Ripoll G., Bertolín J.R., Joy M. (2022). Insights into the Role of Major Bioactive Dietary Nutrients in Lamb Meat Quality: A Review. J. Anim. Sci. Biotechnol..

[13] Iglesias J., Pazos M., Torres J.L., Medina I. (2012). Antioxidant Mechanism of Grape Procyanidins in Muscle Tissues: Redox Interactions with Endogenous Ascorbic Acid and α-Tocopherol. Food Chem..

[14] Rudrapal M., Khairnar S.J., Khan J., Dukhyil A.B., Ansari M.A., Alomary M.N., Alshabrmi F.M., Palai S., Deb P.K., Devi R. (2022). Dietary Polyphenols and Their Role in Oxidative Stress-Induced Human Diseases: Insights Into Protective Effects, Antioxidant Potentials and Mechanism(s) of Action. Front. Pharmacol..

[15] Orzuna-Orzuna J.F., Dorantes-Iturbide G., Lara-Bueno A., Chay-Canul A.J., Miranda-Romero L.A., Mendoza-Martínez G.D. (2023). Meta-Analysis of Flavonoids Use into Beef and Dairy Cattle Diet: Performance, Antioxidant Status, Ruminal Fermentation, Meat Quality, and Milk Composition. Front. Vet. Sci..

[16] Álvarez-Rodríguez J., Villalba D., Molina E., Serrano-Pérez B., Bertolín J.R., Joy M. (2020). ¿Afectan Los Taninos Condensados de La Dieta a Los Resultados Productivos, La Composición de Ácidos Grasos y El Color de La Carne de Cordero?. Inf. Tec. Econ. Agrar..

[17] Frutos P., Hervás G., Natalello A., Luciano G., Fondevila M., Priolo A., Toral P.G. (2020). Ability of Tannins to Modulate Ruminal Lipid Metabolism and Milk and Meat Fatty Acid Profiles. Anim. Feed Sci. Technol..

[18] Pelegrin-Valls J., Serrano-Pérez B., Villalba D., Molina E., Espinal J., Joy M., Álvarez-Rodríguez J. (2022). Is the Inclusion of Carob (*Ceratonia siliqua* L.) Pulp in the Concentrate of Weaned Light Lambs Worth It?. Anim. Feed Sci. Technol..

[19] Bottegal D.N., Lobón S., Latorre M.Á., Bertolín J.R., Álvarez-Rodríguez J. (2023). Colour Stability, Fatty Acid Profile, and Lipid Oxidation in Meat Stored in Modified Atmosphere Packaging from Light Lambs Fed with Concentrate with Carob Pulp (*Ceratonia siliqua* L.). Antioxidants.

[20] Ioannou G.D., Savva I.K., Christou A., Stavrou I.J., Kapnissi-Christodoulou C.P. (2023). Phenolic Profile, Antioxidant Activity, and Chemometric Classification of Carob Pulp and Products. Molecules.

[21] Zhou B., Wu L.M., Yang L., Liu Z.L. (2005). Evidence for α-Tocopherol Regeneration Reaction of Green Tea Polyphenols in SDS Micelles. Free Radic. Biol. Med..

[22] Jerónimo E., Soldado D., Sengo S., Francisco A., Fernandes F., Portugal A.P.V., Alves S.P., Santos-Silva J., Bessa R.J.B. (2020). Increasing the α-Tocopherol Content and Lipid Oxidative Stability of Meat through Dietary *Cistus Ladanifer* L. in Lamb Fed Increasing Levels of Polyunsaturated Fatty Acid Rich Vegetable Oils. Meat Sci..

[23] González-Calvo L., Ripoll G., Molino F., Calvo J.H., Joy M. (2015). The Relationship between Muscle α-Tocopherol Concentration and Meat Oxidation in Light Lambs Fed Vitamin E Supplements Prior to Slaughter. J. Sci. Food Agric..

[24] Álvarez I., De La Fuente J., Díaz M.T., Lauzurica S., Pérez C., Cañeque V. (2008). Estimation of α-Tocopherol Concentration Necessary to Optimise Lamb Meat Quality Stability during Storage in High-Oxygen Modified Atmosphere Using Broken-Line Regression Analysis. Animal.

[25] Bottegal D.N., Lobon S., Serrano-Pérez B., Martín-Alonso M.J., Latorre M.Á., Alvarez-Rodriguez J. (2024). Mild Synergistic Effects of a Dietary Source of Polyphenols (*Ceratonia siliqua* L.) and Vitamin E on Light Lambs’ Rumination Activity, Nutritional Status, and Gastrointestinal Redox-Immune Markers. Livest. Sci..

[26] Rufino-Moya P.J., Blanco M., Bertolín J.R., Joy M. (2019). Effect of the Method of Preservation on the Chemical Composition and in Vitro Fermentation Characteristics in Two Legumes Rich in Condensed Tannins. Anim. Feed Sci. Technol..

[27] Firestone D., American Oil Chemists’ Society (AOCS) (2017). Official Method Am 5-04. Rapid determination of oil/fat utilizing high-temperature solvent extraction. Official Methods and Recommended Practices.

[28] Sukhija P.S., Palmquist D.L. (1988). Rapid Method for Determination of Total Fatty Acid Content and Composition of Feedstuffs and Feces. J. Agric. Food Chem..

[29] Baila C., Joy M., Bertolín J.R., Blanco M., Casasús I., Lobón S. (2023). Effect of Sainfoin Proanthocyanidins on Milk Fatty Acids from Ewes Rearing Suckling Lambs. Animal.

[30] (2015). Animal and Vegetables Fat and Oils. Gas Chromatography of Fatty Acid Methyl Esters. Part 4: Determination by Capillary Chromatography.

[31] Scerra M., Bognanno M., Foti F., Caparra P., Cilione C., Mangano F., Natalello A., Chies L. (2022). Influence of Almond Hulls in Lamb Diets on Animal Performance and Meat Quality. Meat Sci..

[32] Blanco M., Ripoll G., Casasús I., Bertolín J.R., Joy M. (2019). Carotenoids and Tocopherol in Plasma and Subcutaneous Fat Colour to Trace Forage-Feeding in Growing Steers. Livest. Sci..

[33] (2013). Microbiology of the Food Chain—Horizontal Method for the Enumeration of Microorganisms.

[34] (2019). Microbiology of the Food Chain—Horizontal Method for the Enumeration of Psychrotrophic Microorganisms.

[35] Noor-Ehsan Gobindram M.N., Bognanno M., Luciano G., Lanza M., Biondi L. (2015). Carob Pulp Inclusion in Lamb Diets: Effect on Intake, Performance, Feeding Behaviour and Blood Metabolites. Anim. Prod. Sci..

[36] Jacondino L.R., Poli C.H.E.C., Tontini J.F., Corrêa G.F., Somacal S., Mello R.O., Leal M.L.R., Raimondo R.F.S., Riet-Correa B., Muir J.P. (2022). Acacia Mearnsii Tannin Extract and α-Tocopherol Supplementation in Lamb Diet: Effects on Growth Performance, Serum Lipid Peroxidation and Meat Quality. Anim. Feed Sci. Technol..

[37] Ripoll G., González-Calvo L., Molino F., Calvo J.H., Joy M. (2013). Effects of Finishing Period Length with Vitamin E Supplementation and Alfalfa Grazing on Carcass Color and the Evolution of Meat Color and the Lipid Oxidation of Light Lambs. Meat Sci..

[38] Bellés M., del Mar Campo M., Roncalés P., Beltrán J.A. (2019). Supranutritional Doses of Vitamin E to Improve Lamb Meat Quality. Meat Sci..

[39] Facciolongo A.M., Lestingi A., Colonna M.A., Nicastro F., De Marzo D., Toteda F. (2018). Effect of Diet Lipid Source (Linseed vs. Soybean) and Gender on Performance, Meat Quality and Intramuscular Fatty Acid Composition in Fattening Lambs. Small Rumin. Res..

[40] de Araújo T.L.A.C., Pereira E.S., Mizubuti I.Y., Campos A.C.N., Pereira M.W.F., Heinzen E.L., Magalhães H.C.R., Bezerra L.R., da Silva L.P., Oliveira R.L. (2017). Effects of Quantitative Feed Restriction and Sex on Carcass Traits, Meat Quality and Meat Lipid Profile of Morada Nova Lambs. J. Anim. Sci. Biotechnol..

[41] Capcanari T., Covaliov E., Chirsanova A., Popovici V., Radu O., Siminiuc R. (2023). Bioactive Profile of Carob (*Ceratonia siliqua* L.) Cultivated in European and North Africa Agrifood Sectors. Ukr. Food J..

[42] Priolo A., Lanza M., Biondi L., Pappalardo P., Young O.A. (1998). Effect of Partially Replacing Dietary Barley with 20% Carob Pulp on Post-Weaning Growth, and Carcass and Meat Characteristics of Comisana Lambs. Meat Sci..

[43] Gravador R.S., Luciano G., Jongberg S., Bognanno M., Scerra M., Andersen M.L., Lund M.N., Priolo A. (2015). Fatty Acids and Oxidative Stability of Meat from Lambs Fed Carob-Containing Diets. Food Chem..

[44] Gómez-Cortés P., Domenech F.R., Rueda M.C., de la Fuente M.Á., Schiavone A., Marín A.L.M. (2021). Odd-and Branched-Chain Fatty Acids in Lamb Meat as Potential Indicators of Fattening Diet Characteristics. Foods.

[45] Vasta V., Makkar H.P.S., Mele M., Priolo A. (2009). Ruminal Biohydrogenation as Affected by Tannins in Vitro. Br. J. Nutr..

[46] Natalello A., Luciano G., Morbidini L., Valenti B., Pauselli M., Frutos P., Biondi L., Rufino-Moya P.J., Lanza M., Priolo A. (2019). Effect of Feeding Pomegranate Byproduct on Fatty Acid Composition of Ruminal Digesta, Liver, and Muscle in Lambs. J. Agric. Food Chem..

[47] Watkins P.J., Jaborek J.R., Teng F., Day L., Castada H.Z., Baringer S., Wick M. (2021). Branched Chain Fatty Acids in the Flavour of Sheep and Goat Milk and Meat: A Review. Small Rumin. Res..

[48] Berthelot V., Broudiscou L., Schmidely P. (2014). Effect of Vitamin E Supplementation on Fatty Acid Composition of Muscle and Adipose Tissues of Indoor Lambs with Special Attention on Rumen-Derived Trans Monounsaturated Fatty Acids. Meat Sci..

[49] Mapiye C., Dugan M.E.R., Juá Rez M., Basarab J.A., Baron V.S., Turner T., Yang X., Aldai N., Aalhus J.L. (2012). Influence of α-Tocopherol Supplementation on Trans-18:1 and Conjugated Linoleic Acid Profiles in Beef from Steers Fed a Barley-Based Diet. Animal.

[50] Pottier J., Focant M., Debier C., De Buysser G., Goffe C., Mignolet E., Froidmont E., Larondelle Y. (2006). Effect of Dietary Vitamin E on Rumen Biohydrogenation Pathways and Milk Fat Depression in Dairy Cows Fed High-Fat Diets. J. Dairy Sci..

[51] González-Calvo L., Dervishi E., Joy M., Sarto P., Martin-Hernandez R., Serrano M., Ordovás J.M., Calvo J.H. (2017). Genome-Wide Expression Profiling in Muscle and Subcutaneous Fat of Lambs in Response to the Intake of Concentrate Supplemented with Vitamin E. BMC Genom..

[52] Salvatori G., Pantaleo L., Di Cesare C., Maiorano G., Filetti F., Oriani G. (2004). Fatty Acid Composition and Cholesterol Content of Muscles as Related to Genotype and Vitamin E Treatment in Crossbred Lambs. Meat Sci..

[53] Vincenti F., Giusti A.M., Danieli P.P., Ronchi B., Perer F., Macone A., Filippi E., Iacurto M. (2016). Influence of Dietary Vitamin E Supplementation on Cholesterol Oxidation and Fresh Colour in Beef Aged for 3 and 14 Days. Ital. J. Anim. Sci..

[54] Horcada A., Beriain M.J., Purroy A., Lizaso G., Chasco J. (1998). Effect of Sex on Meat Quality of Spanish Lamb Breeds (Lacha and Rasa Aragonesa). Anim. Sci..

[55] Valenti B., Natalello A., Vasta V., Campidonico L., Roscini V., Mattioli S., Pauselli M., Priolo A., Lanza M., Luciano G. (2019). Effect of Different Dietary Tannin Extracts on Lamb Growth Performances and Meat Oxidative Stability: Comparison between Mimosa, Chestnut and Tara. Animal.

[56] Thies A.J., Altmann B.A., Countryman A.M., Smith C., Nair M.N. (2024). Consumer Willingness to Pay (WTP) for Beef Based on Color and Price Discounts. Meat Sci..

[57] Francisco A., Dentinho M.T., Alves S.P., Portugal P.V., Fernandes F., Sengo S., Jerónimo E., Oliveira M.A., Costa P., Sequeira A. (2015). Growth Performance, Carcass and Meat Quality of Lambs Supplemented with Increasing Levels of a Tanniferous Bush (*Cistus ladanifer* L.) and Vegetable Oils. Meat Sci..

[58] Wu H., Bak K.H., Goran G.V., Tatiyaborworntham N. (2024). Inhibitory Mechanisms of Polyphenols on Heme Protein-Mediated Lipid Oxidation in Muscle Food: New Insights and Advances. Crit. Rev. Food Sci. Nutr..

[59] Ponnampalam E.N., Butler K.L., McDonagh M.B., Jacobs J.L., Hopkins D.L. (2012). Relationship between Muscle Antioxidant Status, Forms of Iron, Polyunsaturated Fatty Acids and Functionality (Retail Colour) of Meat in Lambs. Meat Sci..

[60] Lauzurica S., De La Fuente J., Díaz M.T., Álvarez I., Pérez C., Cañeque V. (2005). Effect of Dietary Supplementation of Vitamin e on Characteristics of Lamb Meat Packed under Modified Atmosphere. Meat Sci..

[61] Luan J., Jin Y., Zhang T., Feng X., Geng K., Zhang M., Geng C. (2023). Effects of Dietary Vitamin E Supplementation on Growth Performance, Slaughter Performance, Antioxidant Capacity and Meat Quality Characteristics of Finishing Bulls. Meat Sci..

[62] Vieira C., Guerra-Rivas C., Martínez B., Rubio B., Manso T. (2022). Effects of Grape Pomace Supplementation on the Diet of Lactating Ewes as Compared to Vitamin E on the Meat Shelf Life of Suckling Lambs. Meat Sci..

[63] Guerra-Rivas C., Vieira C., Rubio B., Martínez B., Gallardo B., Mantecón A.R., Lavín P., Manso T. (2016). Effects of Grape Pomace in Growing Lamb Diets Compared with Vitamin E and Grape Seed Extract on Meat Shelf Life. Meat Sci..

[64] Ortuño J., Serrano R., Bañón S. (2015). Antioxidant and Antimicrobial Effects of Dietary Supplementation with Rosemary Diterpenes (Carnosic Acid and Carnosol) vs. Vitamin E on Lamb Meat Packed under Protective Atmosphere. Meat Sci..

[65] Biondi L., Randazzo C.L., Russo N., Pino A., Natalello A., Van Hoorde K., Caggia C. (2019). Dietary Supplementation of Tannin-Extracts to Lambs: Effects on Meat Fatty Acids Composition and Stability and on Microbial Characteristics. Foods.

[66] Meziani S., Oomah B.D., Zaidi F., Simon-Levert A., Bertrand C., Zaidi-Yahiaoui R. (2015). Antibacterial Activity of Carob (*Ceratonia siliqua* L.) Extracts against Phytopathogenic Bacteria Pectobacterium Atrosepticum. Microb. Pathog..

[67] Ibrahim A.H., El-Baky R.M.A., Desoukey S.Y., Abd-Lateff A., Kamel M.S. (2013). Bacterial Growth Inhibitory Effect of *Ceratonia siliqua* L. Plant Extracts Alone and in Combination with Some Antimicrobial Agents. J. Adv. Biotechnol. Bioeng..

[68] Tassou C.C., Drosinos E.H., Nychas G.J.E. (1997). Weak Antimicrobial Effect of Carob (*Ceratonia siliqua*) Extract against Food-Related Bacteria in Culture Media and Model Food Systems. World J. Microbiol. Biotechnol..

