# Supplementary material for: Dietary Inclusion of Carob Pulp (Ceratonia siliqua L.) Does Not Replace the Antioxidant Effect of Vitamin E in Lambs’ Meat to Lengthen Shelf-Life
Source: Animals (Basel). 2024 Dec 16;14(24):3629. doi: 10.3390/ani14243629 (PMC11672404; doi:10.3390/ani14243629)
Supplement: Supplementary file 1 [file animals-14-03629-s001.zip › animals-3299937-supplementary.pdf]

animals

Supplementary material

Supplementary materials

Table S1. Ingredients and chemical composition of experimental diets, with two levels of carob pulp (Cp, 0 vs. 20%) and two doses of vitamin E (Vit E, 40 vs. 300 IU/kg).

<table>
<tr><th>Item</th><th>0% Cp-Low Vit E</th><th>0% Cp-High Vit E</th><th>20% Cp-Low Vit E</th><th>20% Cp-High Vit E</th></tr>
<tr><td>Ingredients (g/kg of feed)</td><td></td><td></td><td></td><td></td></tr>
<tr><td>Carob pulp</td><td colspan="2">0</td><td colspan="2">200</td></tr>
<tr><td>Corn</td><td colspan="2">204</td><td colspan="2">267</td></tr>
<tr><td>Barley</td><td colspan="2">361</td><td colspan="2">150</td></tr>
<tr><td>Wheat</td><td colspan="2">50</td><td colspan="2">50</td></tr>
<tr><td>Soybean husk</td><td colspan="2">150</td><td colspan="2">50</td></tr>
<tr><td>Soy 47%</td><td colspan="2">198</td><td colspan="2">235</td></tr>
<tr><td>Palm oil</td><td colspan="2">2</td><td colspan="2">18.5</td></tr>
<tr><td>CaCO3</td><td colspan="2">25.4</td><td colspan="2">20</td></tr>
<tr><td>Salt</td><td colspan="2">5</td><td colspan="2">5</td></tr>
<tr><td>Vitamin-mineral premix</td><td colspan="2">4</td><td colspan="2">4</td></tr>
<tr><td>Vitamin E 50% all-rac-alpha-tocopheryl acetate</td><td>0</td><td>0.5</td><td>0</td><td>0.5</td></tr>
<tr><td>Nutrients (g/kg of DM feed)</td><td></td><td></td><td></td><td></td></tr>
<tr><td>Dry matter</td><td colspan="2">890</td><td colspan="2">885</td></tr>
<tr><td>Crude protein</td><td colspan="2">173</td><td colspan="2">175</td></tr>
<tr><td>Ether Extract</td><td colspan="2">25.6</td><td colspan="2">33.6</td></tr>
<tr><td>Crude fibre</td><td colspan="2">76.9</td><td colspan="2">54.3</td></tr>
<tr><td>Total sugar</td><td colspan="2">24.7</td><td colspan="2">96.0</td></tr>
<tr><td>Neutral Detergent Fibre</td><td colspan="2">182</td><td colspan="2">178</td></tr>
<tr><td>Acid Detergent Fibre</td><td colspan="2">101</td><td colspan="2">80</td></tr>
<tr><td>Acid-Detergent Lignin</td><td colspan="2">0.88</td><td colspan="2">2.44</td></tr>
<tr><td>Starch</td><td colspan="2">458</td><td colspan="2">387</td></tr>
<tr><td>Ash</td><td colspan="2">55.9</td><td colspan="2">63.4</td></tr>
<tr><td>Calcium</td><td colspan="2">12.8</td><td colspan="2">13.2</td></tr>
<tr><td>Phosphorus</td><td colspan="2">3.26</td><td colspan="2">4.57</td></tr>
<tr><td>Gross energy (MJ/kg DM basis)</td><td colspan="2">17.9</td><td colspan="2">18.8</td></tr>
</table>
